# Embryological, anatomical and clinical considerations on pleuroperitoneal communication

**DOI:** 10.1515/pp-2023-0013

**Published:** 2023-06-19

**Authors:** Christodoulos Chatzigrigoriadis, Anastasios Goulioumis, Despoina Sperdouli, Kostis Gyftopoulos

**Affiliations:** School of Medicine, University of Patras, Patras, Greece; Karamandaneion Paediatric Hospital, Patras, Greece; Department of Anatomy, School of Medicine, University of Patras, Patras, Greece

**Keywords:** communication, peritoneum, pleura, pleuroperitoneal, review

## Abstract

The pleural and peritoneal cavity share many related features due to their common celomic origin. Normally these two spaces are completely separated with the development of the diaphragm. Defects in diaphragm morphogenesis may result in congenital diaphragmatic hernias, which is the most known form of communication between the pleural and peritoneal cavity. However, in several cases, findings of pleuroperitoneal communication (PPC) have been described in adults through an apparently intact diaphragm. In this comprehensive review we systematically evaluate clinical scenarios of this form of “unexpected” PPC as reported in the literature and focus on the possible mechanisms involved.

## Introduction

Peritoneal and pleural cavities are considered to be separated since fetal life, with the development of the diaphragm. Defects in diaphragm morphogenesis may result in congenital diaphragmatic hernias (CDH), which is the most known form of communication between the pleural and peritoneal cavity. The etiology of CDH remains unclear and factors including genetic, environmental, and nutritional defects have been proposed. The majority of the cases (70–75 %) present with an isolated defect, usually at the posterolateral site of the diaphragm (Bochdalek hernias), followed by the less infrequent Morgagni hernias (23–28 %), which present as an anterior defect [1]. In both cases, the herniation of abdominal viscera through the diaphragmatic defect poses an increased risk of interference with the normal development of the lungs (lung hypoplasia/immaturity) and is associated with increased morbidity and mortality [1]. However, several studies have reported the existence of unexpected pleuroperitoneal communication (PPC) that facilitates the transduction of substances, including gas, fluids, cells, tissues, and microbes, from one cavity to the other, through an apparently intact diaphragm. The natural route of this communication is from the peritoneal towards the pleural cavity, due to the pressure gradient between the abdomen and the thorax, although occasionally, the opposite flow can be observed when the pressure gradient is reversed. The exact incidence of this situation is unknown, although in the most common form of PPC occurring in peritoneal dialysis patients is estimated at 2 % [2]. However, sporadic cases of other types of PPC have been described in different clinical scenarios, including inflammatory conditions, hepatic, and gynecological disease.

This review aims to explore the basis of pleuroperitoneal communication (PPC) from the perspective of embryology, histology, and anatomy and the clinical consequences of this communication.

## Embryological considerations

The diaphragm, as a muscle, derives from the mesoderm. The major contributors to the developing diaphragm are the septum transversum, the pleuroperitoneal folds, the mesenchyme ventral and dorsal to the esophagus, and the cervical somites C3–C5 or C2–C6 [3].

**Figure 1:**
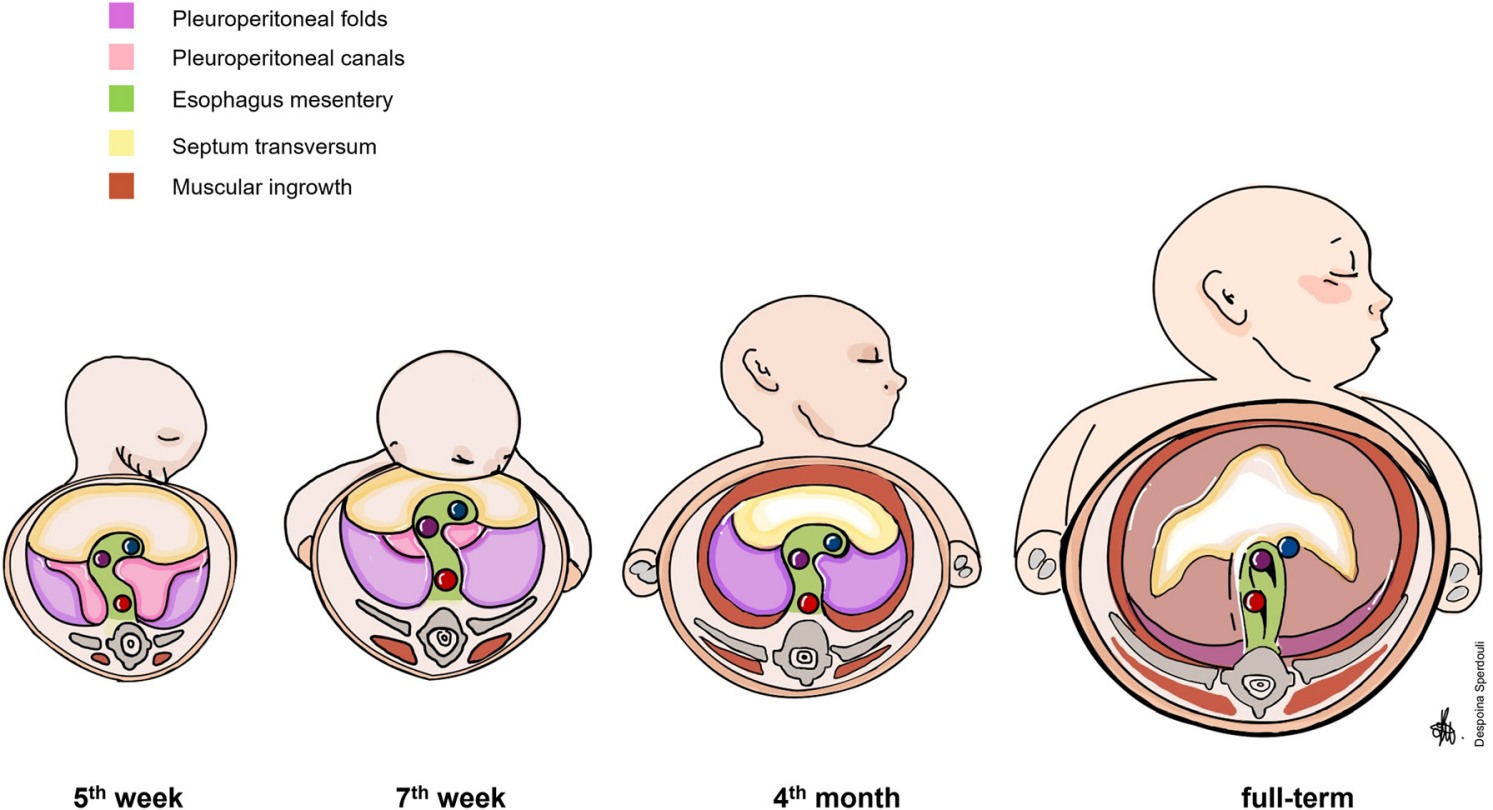
Embryological evolution of the diaphragm.

The septum transversum is a mesodermal component. Anteriorly it fuses with the mesenchyme ventral to the esophagus and participates in the formation of the central tendon of the diaphragm, with the contribution of the pleuroperitoneal folds. The pleuroperitoneal folds are derivatives of the lateral mesoderm, and they are responsible for the development of the muscle connective tissue of the diaphragm. This tissue not only strenghtens the structural architecture of the diaphragm but also serves as a scaffold for the migration of the muscle progenitor cells from the cervical somites [3, 4]. During fetal life, the pleuroperitoneal folds develop dorsolaterally from the body wall to the septum transversum until they fuse with it. They also fuse with the mesenchyme dorsal to the esophagus (Figure 1).

The embryology of the epithelia related to the diaphragm is also of interest. The visceral pleura derive from the splanchnic part of the lateral mesoderm. In contrast, the parietal pleura originate from the parietal part of the lateral mesoderm, similarly to the peritoneum. The endothelium of the diaphragm is associated with the lateral mesoderm; the endothelial and the muscle cells probably share a common cellular ancestor with the somites [5].

There are indications for an embryological basis behind the theory of PPC through lymphatic vessels. This hypothesis is based on the fact that the pleuroperitoneal folds (i.e., the major borders between the peritoneal and the pleural cavities) derive from the lateral mesoderm (like the mesothelium of the cavities and the endothelium of the vessels), and they determine the formation of the diaphragmatic vessels [3]. An attractive hypothesis is whether vessels become a bridge between the peritoneal and the pleural cavities, as discussed later in the histology section.

Notably, there are differences in the development of the right and left hemidiaphragm during morphogenesis. First, the left phrenic nerve appears and distributes later than the right [3]. In addition, the right-side congenital diaphragmatic hernias (CDH), although less frequent, tend to be more prominent in size than the left [6, 7]. However, it should be noted that there is not yet a clear etiopathological relation between these observations. Additionally, both CDH and PPC allow communication between the abdomen and the thorax, but in different locations: the CDH tends to appear in the muscular part of the diaphragm in contrast with the PPC, which is related to the central tendon, the foramina, and the lymphatics [8].

## Anatomical considerations

**Figure 2:**
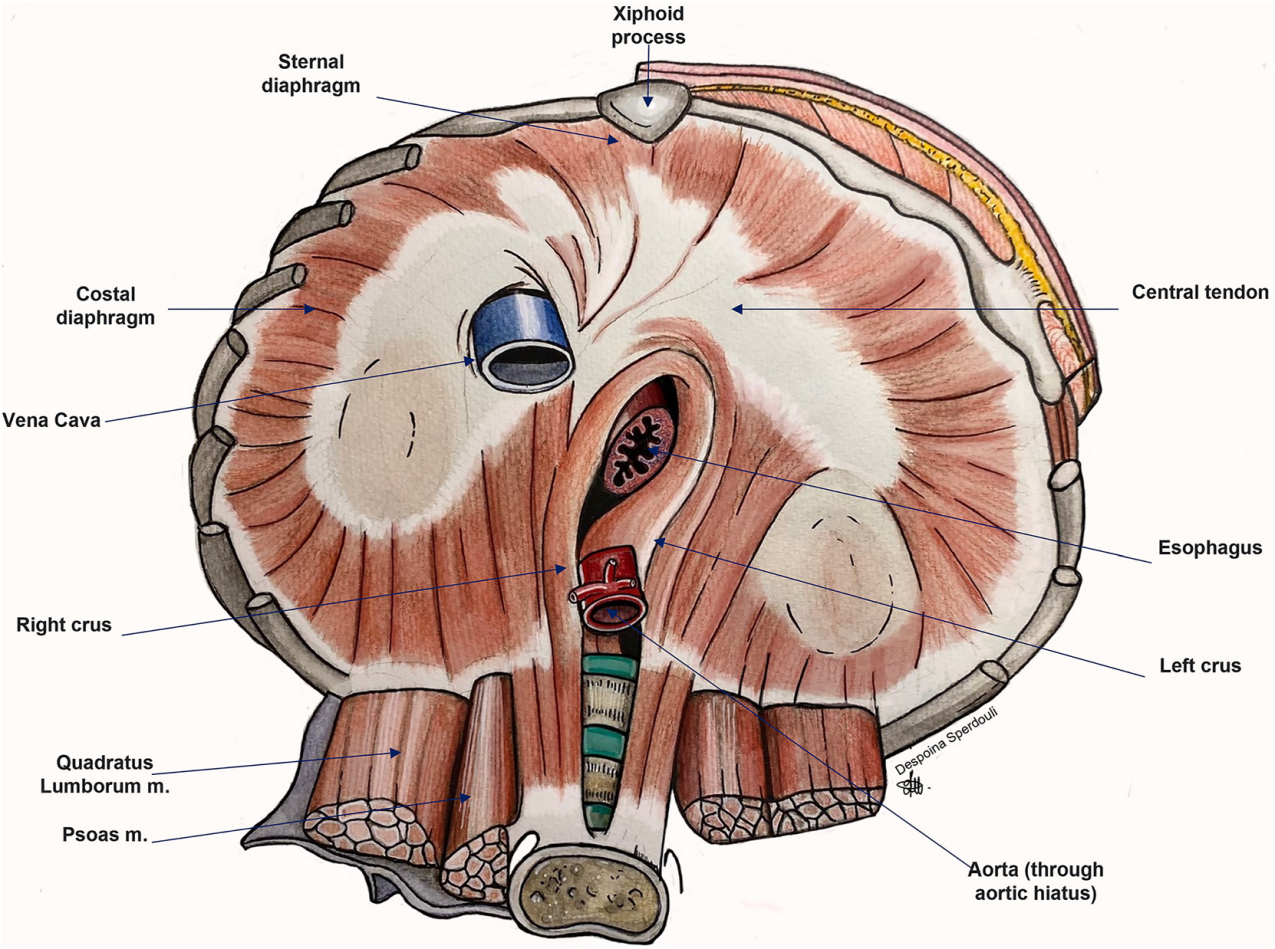
The main components of the adult diaphragm.

The diaphragm is the most important respiratory muscle that also serves as the border between the abdominal and thoracic cavity. It is divided into three main areas: the central tendon, the costal (ventrally), the crural (dorsally) and -less frequently-the sternal diaphragm. The central tendon is a bird-like structure, with the main part located under the pericardium, and serves as an adherence scaffold for the skeletal muscle fibers that radiate to the body wall [9] (Figure 2). The primary nerve responsible for the motor and sensory innervation of the diaphragm is the phrenic nerve. In addition, the lower six (or seven) intercostal nerves and the subpleural nerves contribute to the sensory innervation of the diaphragm. The same phenomenon occurs at the parietal pleura of the diaphragm: the proximal part is innervated by the phrenic nerve and the distal part by the lower five intercostal nerves. Consequently, there is a solid anatomical relation between the diaphragm and the parietal pleura. Notably, there is no relation between the phrenic nerve and the connective tissue of the central tendon.

The lymphatic drainage of the diaphragm is facilitated by the upper and lower surface lymphatics. The lymphatics of the lower surface are characterized by a significant ability to remove fluid from the peritoneal cavity. These two groups of lymphatics inter-communicate, but the lymph normally moves upwards as the intra-abdominal pressure overcomes the intrathoracic pressure. This type of communication could partially explain a form of PPC [10].

Anatomical differences between the right and the left hemidiaphragm may be responsible for particular types of pleuroperitoneal communication. The left hemidiaphragm is lower than the right hemidiaphragm, as it is pushed downwards by the pericardial sac, while the right hemidiaphragm is displaced upwards by the liver. Thus, the left hemidiaphragm is mainly covered by the fibrous pericardium, and the right hemidiaphragm is domed over the liver. The right and the left subphrenic space lie between the right hemidiaphragm and the liver and are divided by the falciform ligament, which is attached to the diaphragm and the anterior abdominal wall [11, 12]. The bare area (*area nuda hepatis*) of the liver is a triangular area located at the posterosuperior surface of the right lobe of the liver; it lies between the two layers of the coronary ligament (upper and lower) and the right triangular ligament. It is the only area of the liver devoid of visceral peritoneum covering and is separated by thin multilayered connective tissue from the diaphragm [13]. Thus, the right hemidiaphragm is in closer contact with the liver than the left hemidiaphragm does with the stomach or the spleen, respectively. Moreover, the right hemidiaphragm is mechanically less resistant than the left because it is less thick and muscular [3, 14]. The porous diaphragm syndrome (gaps in the diaphragm that allow PPC commonly seen in the central tendon of the diaphragm) tends to appear on the right rather than the left side [15]. Finally, the lymph drainage from the right hemidiaphragm is more efficient than the drainage from the left hemidiaphragm [16]. In contrast, there is no difference between the right and left pulmonary ligaments’ lymph drainage [17].

## Histological considerations

The pleural and the peritoneal cavity are covered by the mesothelium, which forms both the visceral and the parietal pleura and peritoneum, respectively. Typically, the mesothelium is considered a simple squamous epithelium characterized by microvilli projecting to the cavity (i.e., microvilli facing the apical surface). However, a small part of the mesothelium consists of cuboidal cells. This difference in the mesothelial cell structure may affect the cavities’ lymphatic drainage [16], [17], [18], [19], [20], [21], [22]. The mesothelium, like any other epithelium, is supported by the basement membrane over a layer rich in elastic fibers. Underneath the basal lamina, layers of elastic lamina, loose connective tissue (where the lymphatics are located), and finally, a fibro-elastic fiber layer are present [19]. The balance between production and removal of fluid maintains the equilibrium in these cavities. Normally, the blood capillaries filter the serous fluid in these cavities and supply the mesothelium and the supportive connective tissue with oxygen and nutrients. This filtration is regulated by Starling’s law: the hydrostatic pressure gradient tends to move water out of the capillaries and in contrast, the colloid osmotic pressure gradient tends to move water inside the capillaries. The filtration rate depends on the permeability of the capillaries in the water and the plasma proteins, which eventually determine the colloid osmotic pressure. Growth factors, like the vascular endothelial growth factor (VEGF), contribute significantly to the increase of capillary permeability [19]. Under normal conditions, a slight excess of fluid is produced by this process. Hence, the lymphatic cleavage is responsible for maintaining the fluid volume in the cavities, by removing the excess in both normal and pathological conditions, such as PPC. The lymphatics’ absorbing capacity can be increased by approximately 30 times (at least as it refers to the pleura) [17, 20].

**Figure 3:**
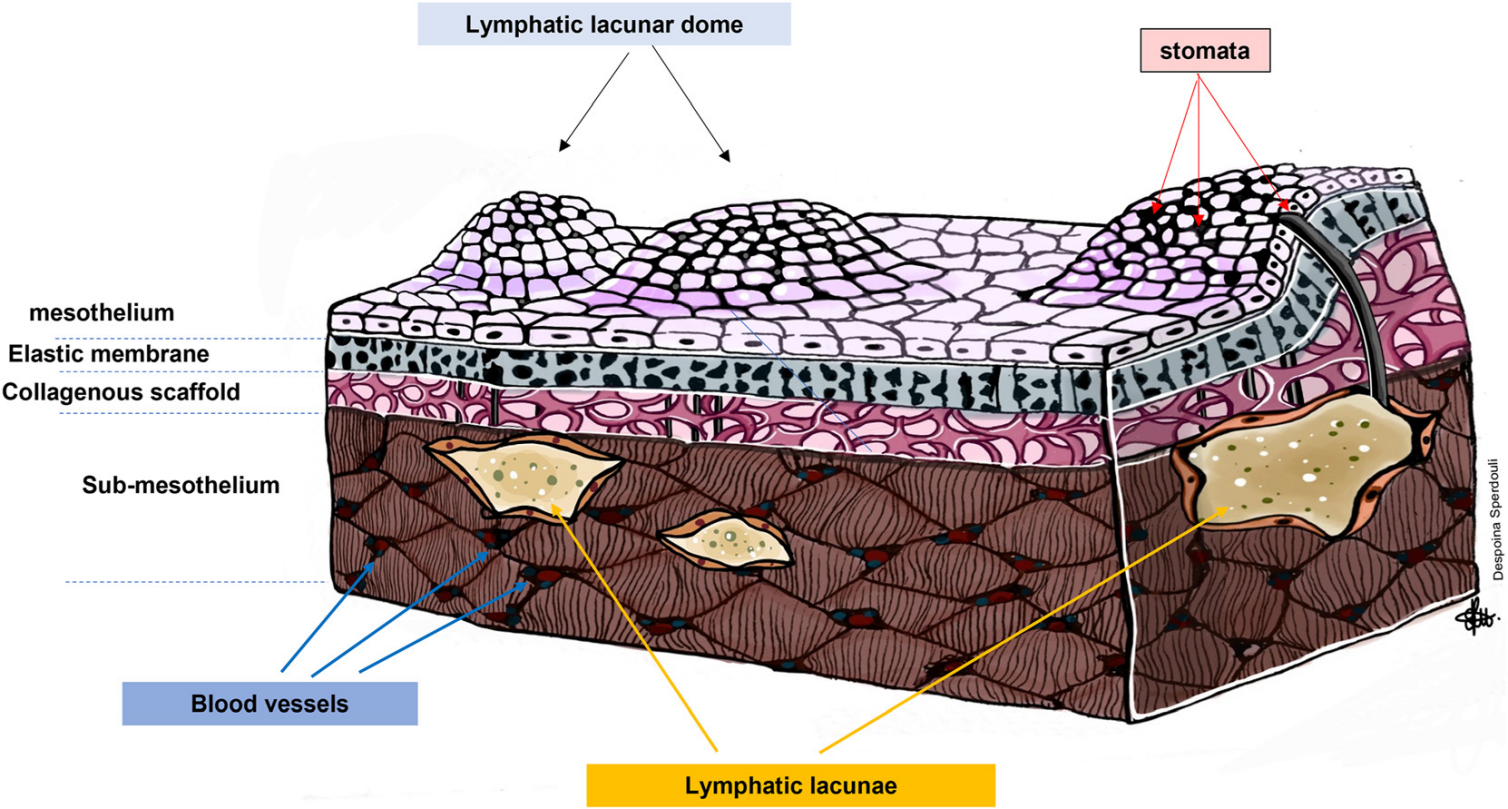
Depiction of the layers of the submesothelial tissue of the diaphragm and the direct communication of the lymphatic stomata with the lymphatic lacunae and drainage system.

The flattened mesothelium is disrupted in some areas by the lymphatic stomata, which are covered (almost always) by cuboidal mesothelium. The lymphatic stomata are a projection of the lymphatics to the cuboidal mesothelium. These gaps communicate with the blind ends of the lymphatic capillaries in the loose connective tissue (also referred to as lymphatic lacunae) through the submesothelial channels (Figure 3). It should be noted that the above mentioned typical three-layered submesothelial tissue, particularly the basement membrane, is absent in the area of the lymphatic stomata [16]. Instead, the fibers of the connective tissue cover these channels. As a result, the peritoneal or the pleural fluid can move from the cavity’s space to the lymphatics’ lumen through the lymphatic stomata, the submesothelial channels, and the blind ends of the lymphatics. Notably, the cuboidal mesothelial and endothelial cells at the beginning of the lymphatics function like valves to prevent lymph regurgitation [16, 18]. However, it has been observed that the cytoplasmic processes of these cells do not fully cover some stomata, so there might be a small degree of regurgitation [17]. Interestingly, the subperitoneal tissue’s lymphatic lacunae are mostly located in the lower surface of the diaphragm and mainly on the right side [10, 15, 16, 18]. Hence, the most significant amount of the peritoneal fluid passes through the lymph vessels of the diaphragm. Their distribution in the diaphragm varies; there are two layers in the muscular part of the diaphragm (the subperitoneal plexus, which anastomoses with the deep plexus) and a single layer (deep plexus only) over the central tendon [16]. The majority of the subpleural lymphatics (85%) is accumulated in the dorsal part of the three or four last intercostal spaces; thus, most of the subpleural lymphatic lacunae are close to the diaphragm and the subperitoneal lymphatic lacunae. Additionally, the lymphatic stomata have been found only in the parietal pleura [20]. These microanatomical characteristics of the lymph pathways could support the theory according to which the PPC is partially mediated by the lymphatics [23].

Although the pleural and peritoneal cavities are considered separate, several reports of PPC are available in the literature. Hydrothorax is more common, although reverse flow from the pleural cavities to the peritoneal cavity has also been observed. In many cases, the mechanisms are multifactorial or undetermined. Below we present several reported clinical entities with pleuroperitoneal communication.

## Peritoneal dialysis (PD) induced hydrothorax

PD-induced hydrothorax is likely the most common form of PPC, observed in approximately 2% of the patients undergoing PD [2, 24], [25], [26], [27], [28], [29], [30], [31]. Congenital and acquired defects, but also the lymphatics of the diaphragm, play a significant role in the pathogenesis of PPC, due to the pressure gradient between the abdomen and thorax [12]. The term “porous diaphragm syndrome” is used to describe symptomatic diaphragmatic defects, which are usually located on the right side of the tendinous diaphragm over the bare area of the liver and most of them are acquired [12, 15, 23, 31–33]. These pores vary from solitary to multiple lesions with variable diameters [2]. Common risk factors include polycystic kidney disease (PKD), peritonitis and systemic amyloidosis, probably due to the weakening of the diaphragmatic tissue [28, 33]. Any cause of intra-abdominal hypertension or infiltration of the diaphragm predisposes to porous diaphragm syndrome. For example, a sharp increase in intra-abdominal pressure (IAP) during Valsalva-like maneuvers (e.g., coughing) and a moderate increase during PD predispose for porous diaphragm syndrome [34]. When the intra-abdominal pressure increases due to the PD dialysate, the pre-existing pores enlarge and become active in a few hours or days, or new pores are formed after some weeks or months [28]. A cut-off value of 1 month may be considered to classify the porous diaphragm syndrome as congenital or acquired [33].

Another mechanism of PD-induced pleural effusion involves the lymphatic stomata in the serosal surfaces of the diaphragm [23]. These microscopic holes allow the passage of the peritoneal fluid into the lymphatic lacunae (lymphatic capillaries of the diaphragm) on the way to the pleural cavities. The peritoneal stomata are located exclusively in the diaphragm [18], while the lymphatic lacunae are found only in the muscular portion of the diaphragm, mainly on the right side [16]. In addition, the pleural stomata are observed mainly in the lower portion of the pleura and their position may explain the intercommunication between the two cavities [20]. The lymphatics in the muscular portion determine the baseline absorption rate of the peritoneal fluid while the tendinous lymphatics, which are more abundant, determine the higher absorption rate in ascites or PD dialysate [35]. These data support that the lymph plexus of the diaphragm is a dynamic structure, serving the transport of the PD dialysate from the peritoneal cavity to the pleural cavity (usually on the right side).

The flow of the PD dialysate from the abdomen to the thorax is presented mainly as a right rather than a left pleural effusion. Occasionally, PD is complicated by right and left pleural effusion simultaneously [28]. This preference for the right side is multifactorial. The pericardial sac tamponades the left hemidiaphragm preventing a left pleural effusion, even in the presence of a left defect [28, 33]. The intestinal peristaltic waves by the ascending colon push the fluid from the pelvis and the abdomen to the right subphrenic space through the right paracolic gutter [11, 15]. The right subphrenic space is located between the liver and the right hemidiaphragm and is separated from the left subphrenic space by the falciform ligament [36]. The fluid collection in the right-subphrenic space is increased during the inspiration, due to the expansion of the rib cage [15]. At the same time, the right hemidiaphragm presses on the liver surface, creating a “piston effect” between the liver and the diaphragm, which significantly increases the hydrostatic pressure in the right subphrenic area during the inspiration [15]. On the contrary, these forces do not develop in the left upper quadrant due to the soft surface of the stomach and the spleen and the lack of peristaltic waves or a bounder such as the falciform ligament [15]. Moreover, the pores and lymphatics are usually located at the right hemidiaphragm [15, 16, 23]. Thus, the relatively high pressure and absorption rate in the right hemidiaphragm and the more frequent location of acquired pores and lymphatic lacunae on the right side of the diaphragm, may explain the tendency for right sided hydrothorax in PD patients.

## Hepatic hydrothorax

Another well described presentation of PPC is hepatic hydrothorax. Hepatic hydrothorax refers to pleural effusion in cirrhotic patients due to the movement of peritoneal fluid in the pleural cavities (usually on the right side). It is observed in at least 5 % of patients with cirrhosis [36], [37], [38]. Ascites is present in most of these patients, but it is not required to establish the diagnosis of hepatic hydrothorax [36], [37], [38]. The pathogenesis of hepatic hydrothorax involves the usual mechanisms of edema in patients with end-stage liver disease, such as hypoalbuminemia and azygos vein hypertension [36]. Nevertheless, these mechanisms fail to explain why the transudate accumulates in the right pleural cavity. Thus, congenital or acquired diaphragmatic defects, the diaphragm’s lymph plexus, the intestinal peristalsis, the piston effect of the liver, the respiratory cycle, and the pericardial sac should be taken into consideration, as in PD-induced hydrothorax. All these different mechanisms may explain the absence of macroscopic diaphragmatic defects in patients with hepatic hydrothorax [38]. The presence of ascites certainly predisposes to hepatic hydrothorax, as high intra-abdominal pressure increases the lymph flow and the risk for porous diaphragm syndrome, especially in primary bacterial peritonitis [21, 35], [36], [37]. Hepatic hydrothorax in the absence of ascites occurs when the peritoneal fluid passes into the pleural cavities without accumulating in the peritoneal cavity [36]. Hepatic hydrothorax without ascites should raise suspicion for congenital porous diaphragm syndrome or other causes of acquired diaphragmatic defects such as Valsalva maneuver, trauma, and surgery [36, 38].

## Peritonitis

Inflammation of the serosal cavities contributes to the remodeling of the peritoneum, the pleura and the diaphragm, increasing the risk of acquired PPC. It is well established that peritonitis is a risk factor for acquired porous diaphragm syndrome, as mentioned above [28, 33]. This syndrome is probably attributable to inflammatory cells which release enzymes that digest the tissues and inflammatory cytokines that activate catabolic processes in the muscle fibers, such as proteolysis. Furthermore, it has been observed that the number and diameter of the lymphatic stomata in the diaphragm increase after peritonitis [21]. In addition, inflammation increases the permeability of the pleural and peritoneal vessels through VEGF and other inflammatory cytokines, contributing to the accumulation of exudate in these cavities [19]. The dynamics of lymph and blood vessels may explain why the flow of the peritoneal fluid to the pleural cavities increases after inflammation, especially in the presence of ascites and high abdominal pressure. However, it must be noted that peritonitis can lead to purulent pleural effusion (combined with pneumothorax in the presence of perforation) due to pre-existing PPC [39]. A case of tuberculous pleuritis has been reported due to tuberculous peritonitis as a complication of PD in the presence of PPC [23]. Another case of tuberculous empyema due to pyelonephritis has been reported, which was treated with the correction of PPC and antibiotics [40]. These cases emphasize the possible spread of *M. tuberculosis* through the diaphragm.

A particular case of peritonitis presenting with PPC is pseudo-pseudo-Meigs syndrome (PPMS) or Tjalma syndrome [41, 42]. PPMS is an infrequent complication of systemic lupus erythematosus (SLE) which presents with the triad of ascites, pleural effusion, and elevated CA-125 in patients with SLE. In this case, ascitic fluid is a non-infectious exudate. Elevation of CA-125 can be attributed to the inflammation of the peritoneum, but this relationship has yet to be elucidated. Interestingly, inflammatory cytokines such as IL-1, IL-6, and VEGF activate the peritoneum, as observed in other causes of serositis [41].

## Gynecologic diseases

PPC may be associated with many gynecologic conditions. Common examples include ovarian fibromas and other neoplasms (either benign or malignant), endometriosis, and ectopic pregnancy.

Meigs syndrome is the triad of the ovarian fibroma (or thecoma, granulosa cell tumor, Brenner tumor), ascites, and pleural effusion [43, 44]. Pseudo-Meigs syndrome is the presence of an ovarian or pelvic tumor (either benign or malignant), ascites, and pleural effusion [45]. Meigs and pseudo-Meigs syndrome share a common pathophysiology and clinical presentation but differ in primary etiology and treatment. Ovarian fibroma in Meigs syndrome is a benign ovarian neoplasm. Instead, the pseudo-Meigs syndrome has been associated with uterine leiomyoma, primary and metastatic malignant ovarian neoplasm due to gastric, breast, and colorectal cancer [45]. Two theories have been proposed for the pathogenesis of ascites in both syndromes. The first theory notes a mismatch between arterial blood flow and venous blood/lymph flow, which depends on the size of the neoplasm and produces a transudate [43, 45]. Further research should evaluate the role of lymphatic stomata in the ovarian bursa, as it refers to the production and flow of oedematous fluid from the neoplasm to the peritoneal cavity [20]. The second theory suggests peritoneal irritation by the neoplasm as the main mechanism [43, 45]. VEGF plays a significant role in this process by inducing the formation of new vessels and increasing their permeability [19, 45]. The peritoneum produces increased amounts of fluid, contributing to ascites formation. This theory explains the elevation of CA 125 in these patients, given that the activated mesothelial cells secrete CA 125, as mentioned above [43]. The presence of pleural effusion is required for the diagnosis of Meigs or pseudo-Meigs syndrome. Given that the malignant neoplastic cells do not infiltrate the diaphragm, another form of PPC should be postulated [43]. Mechanisms already mentioned above, such as pores, lymphatics, intestinal peristalsis, the piston effect of the liver, the respiratory movement of the rib cage, and the pericardial barrier, could explain the fact that rarely these patients present with left pleural effusion [45]. However, it must be noted that PPC through lymphatics is not universally accepted [43]. Some authors propose that the peritoneal fluid moves through the normal foramina (for the inferior vena cava, the esophagus, and the aorta) of the diaphragm [43].

Ovarian hyperstimulation syndrome is classified together with the Meigs and the pseudo-Meigs syndrome, as it refers to a similar pathophysiology and clinical presentation. The same mechanisms are implicated in the pathogenesis of ascites and pleural effusion in ovarian hyperstimulation syndrome [44].

Another rare clinical presentation of PPC in gynecological patients has been described as hemothorax secondary to a twisted ovarian neoplasm. In this case, a twisted tumor led to red infarct of the ovary, resulting in bloody ascites and concomitant hemothorax, due to a small (5 mm) fenestra, centrally at the right hemidiaphragm [44].

Endometriosis is primarily found in the pelvis and its location in the diaphragm or the thorax is considered rare [46]. Most cases which affect the diaphragm are asymptomatic because only a portion of the muscle is involved, while chest X-ray is often normal [46]. In more severe cases, invasive endometrium may result in catamenial hemoptysis, hemopericardium, hemothorax, and pneumothorax, depending on the exact location (lung, pericardium, and pleural cavities, respectively) [46, 47]. Catamenial pneumothorax could also result from air movement from the pelvis and the abdomen to the thorax [47]. Many theories have been proposed to explain the thoracic involvement in endometriosis: metaplasia, transport through the venous system, retrograde flow, and porous diaphragm syndrome [46]. The metaplasia theory is based on the common origin of the peritoneum, pleura, and Mullerian duct derivatives, such as the endometrium, from the celomic epithelium [46]. Consequently, diaphragmatic and thoracic endometriosis could result from celomic metaplasia in the peritoneum or pleura. In addition, the endometrium may migrate through the uterine veins and become implanted in the lung parenchyma and pleural cavities [46, 47]. However, these theories fail to explain the right predominance of thoracic endometriosis [46]. The retrograde flow theory supports that the endometrial implants pass through the fallopian tubes into the peritoneal cavity [46] and subsequent transperitoneal–transdiaphragmatic migration of endometrial tissue occurs [48]. As mentioned above, the intestinal peristalsis, the thoracic wall’s inspiratory expansion, and the liver’s piston effect may lead to endometrial implants at the right subphrenic space [7, 47]. Additionally, congenital or acquired porous diaphragm syndrome may facilitate the movement of endometrium in the thorax [15, 46]. The role of lymphatics should not be neglected as a possible pathway from the abdomen to the thorax, given that lymphatic stomata allow the entry of cells [14, 47]. Thus, the theory of retrograde flow is a plausible explanation of the right predominance of endometrial implants in the diaphragm and the pleural cavities [15, 46].

Ectopic pregnancy has rarely been found in the thorax and it has been associated with hemothorax, when found in the pleural cavity. Several mechanisms, including retrograde flow with intestinal peristaltic and respiratory movements and the piston effect of the liver, may lead the embryo under the right hemidiaphragm [15, 46]. The right-predominant porous diaphragm syndrome and the protective presence of the pericardial sac at the left side favor the implantation at the right hemithorax [12, 15, 23, 28, 33].

## Iatrogenic causes

Following endoscopic procedures and abdominal surgery, PPC may be revealed as pleural effusion or pneumothorax. In these cases, PPC either pre-exists or develops during the procedure.

Endoscopic procedures involving the gastrointestinal (GI) system may be complicated by abnormal fluid or air movement from the abdomen to the thorax. A case of acute hydrothorax has been reported in a patient with liver cirrhosis after upper GI endoscopy [49]. This case report represents an acute hepatic hydrothorax caused by the already-mentioned mechanisms and triggered by increased intra-abdominal pressure during the endoscopy. Another case report refers to a patient with acute onset pneumoretroperitoneum, pneumoperitoneum, bilateral pneumothorax, pneumomediastinum, and subcutaneous emphysema [50]. Micro-perforations may occur during endoscopy, leading to the accumulation of air inside the abdominal cavity. The porous diaphragm syndrome was also observed in this patient [50]. These findings suggest that air is free to move between the body cavities when communication points are present, as is observed with fluids and tissues [47].

Pneumothorax is also a rare complication of laparoscopic operations, providing evidence for PPC [26, 51, 52]. PPC can be either pre-existing or may develop during surgery. Pre-existing PPC occurs when the CO_2_ gas passes from the abdomen to the thorax through congenital pores or hiatus of the diaphragm [51, 52]. Acquired PPC occurs when the diaphragm is injured either by the surgeon or by the increased pressure of abdominal gas [51, 52]. The reverse-Trendelenburg position of the patient may contribute to the pathogenesis because the liver and the omentum are positioned downwards, exposing the right hemidiaphragm and its possible defects [52]. The right predominance of the pneumothorax associated with laparoscopic pneumoperitoneum must be noted [52]. Additionally, liver surgery increases the risk for right-sided PPC due to the possibility of accidental diaphragmatic injury.

Pelvic surgery may also be complicated by hemoperitoneum combined with hemothorax due to PPC, as in cases of laparoscopic hysterectomy and myectomy [53, 54]. As described above, PPC can be either congenital or acquired. Laparoscopic pneumoperitoneum is considered to contribute significantly to the creation of PPC [53, 54]. In addition, the hemoperitoneum may result in right-sided pleural effusion through pores and lymphatics, while additional mechanisms involve intestinal peristalsis, respiratory movements, and the piston effect of the liver. These factors and the presence of pericardium on the left explain the right predominance of hemothorax in such cases [15, 53, 54]. Interestingly, the defects in the diaphragm may facilitate the reduction of the accumulated pleural fluid during the postoperative course, suggesting a two-way communication [53]. A case of hepatic hydrothorax triggered by ligation of communicating hydrocele has also been reported [55]. The closure of the *processus vaginalis* was complicated by the accumulation of ascitic fluid, and concomitant decompensated liver cirrhosis, may explain the deterioration of the pleural effusion.

Microwave ablation (MWA) is a novel surgical approach in the treatment of hepatocellular carcinoma (HCC). A rare complication of this technique is pleural effusion due to the development of an acquired PPC. Possible mechanisms include the thermal injury of the diaphragm and the penetration of the diaphragm by the needle during the procedure [56, 57]. The predisposition for right pleural effusion is explained by the fact that the right hemidiaphragm is thinner and less muscular than the left hemidiaphragm [56]. Additionally, the right hemidiaphragm is more exposed than the left hemidiaphragm during the procedure. The already mentioned mechanisms of hepatic hydrothorax may contribute to this complication, but they fail to explain the acute onset of post-MWA pleural effusion [56]. Hence, the role of these mechanisms is questionable in post-MWA pleural effusion.

Chylous ascites rarely causes chylothorax in the presence of PPC. This condition may result from abdominal surgery, such as pancreatectomy [58]. Chylous ascites is considered a rare complication of pancreatic resection due to the injury of the thoracic duct. If the diaphragm is injured during surgery or a pre-existing PPC is present, chylothorax may develop. Chylothorax may also develop in the presence of PPC during parenteral nutrition [59]. If the central venous catheter is not properly positioned, chylous ascites may be formed. As mentioned above, pre-existing PPC becomes active, or new PPC develops, due to the acute elevation of intra-abdominal pressure [59].

## Malignant causes

PPC rarely occurs in patients with cancer and ascites, presenting as an unexplained pleural effusion, usually unilateral. However, the prevalence of pleural effusions associated with ovarian cancer (OC) may be underestimated and underreported. In a recent retrospective study of 189 women with OC, pleural effusion was as high as 43 %, presenting early or during the course of the disease [60]. The pseudo-Meigs syndrome has already been described in patients with gynecological malignancies such as ovarian cancer. Right pleural effusion in a patient with pseudomyxoma peritonei has also been reported [61]. Common mechanisms involve porous diaphragm syndrome and lymphatics, diaphragmatic metastases, and iatrogenic injury of the diaphragm [39]. Pores and lymphatics allow the passage of ascitic fluid and cancer cells into the pleural cavity (usually on the right side), leading to malignant pleural effusion. The role of lymphatics in the transportation of cells has been documented in histologic sections [20]. In addition, cancer cells in the abdominal cavity tend to implant into absorbing surfaces of the peritoneum, such as the diaphragmatic peritoneum, leading to PPC [18, 61]. Surgical management of intra-abdominal malignancies may interrupt the continuity of the diaphragm, thus predisposing for PPC. Right-side predominance may be easily explained by the peritoneal circulation, the piston effect of the liver, the pericardial barrier, and the chest wall movement.

## Lymph circulation abnormalities

The lymph system plays an essential role in the absorption of peritoneal and pleural fluid. It is also involved in the pathogenesis of PPC. Hence, its structural anomalies can present as concomitant chylous ascites and chylothorax.

In a pediatric case report, a patient with generalized lymphatic anomaly (GLA), developed chylous ascites which was complicated by chylothorax [62]. Conservative treatment was ineffective, and the patient had to be treated with perito-neovenous shunt (PVS) placement. The reduction of ascites in this case suggests that PPC was an event secondary to the development of ascites. Due to the late onset of chylothorax during the progression of the disease, acquired PPC should be suspected. The elevated intra-abdominal pressure probably facilitated the acquired PPC.

An additional case of an adult female patient with lymphangioleiomyomatosis (LAM) presenting with chylous ascites and chylothorax has also been reported [63]. A papillary LAM lesion was revealed to connect the peritoneal and the left pleural cavity. Diaphragmatic LAM lesions seem to predispose for direct development of chylothorax [63]. Interestingly, in this case report, bloody chyle was present. Experimental studies suggest that LAM-associated neo-lymphangiogenesis may be facilitated by VEGF-D, which induces aberrant blood-lymphatic vessel formation; this may explain the effusions’ bloody appearance [63, 64]. Of course, porous diaphragm syndrome may contribute to the pathophysiology of chylothorax in the presence of chylous ascites in other patients without diaphragmatic LAM lesions. Moreover, if the LAM affects the pleural and the peritoneal cavities simultaneously, then chylous ascites and chylothorax can occur independently. Table 1 summarizes the prevailing mechanisms of PPC in different clinical scenarios.

**Table 1:** Summary of the prevailing mechanisms of PPC in different clinical scenarios.

Clinical entity	Common mechanisms of PPC
Peritoneal dialysis	Porous diaphragm syndrome (PDS), lymph circulation, retrograde flow (intestinal peristaltism, piston effect of the liver, pericardium)
Hepatic hydrothorax	Hypoalbuminemia, portal hypertension, hyperaldosteronism, PDS, lymph circulation, retrograde flow, spontaneous bacterial peritonitis (SBP)
Peritonitis	Catabolism of the muscle fibers, VEGF-induced remodeling of the lymph circulation, PDS, retrograde flow
Pseudo-pseudo-Meigs syndrome (Tjalma syndrome)	Peritoneal activation (CA-125, VEGF, IL-1, IL-6), PDS, lymph circulation, retrograde flow
Meigs and pseudo-Meigs syndrome Ovarian hyperstimulation syndrome	Arterial-venous mismatch, peritoneal activation, PDS, lymph circulation, retrograde flow
Endometriosis	Metaplasia, venous return, PDS, lymph circulation, retrograde flow
Ectopic pregnancy	PDS, lymph circulation, retrograde flow
Gastrointestinal endoscopic procedures	(Micro)perforation, PDS, lymph circulation, retrograde flow
Laparoscopic surgery	Pneumoperitoneum, PDS, lymph circulation, retrograde flow
Pelvic surgery	Trendelenburg position, PDS, lymph circulation, retrograde flow
Microwave ablation of hepatocellular Ca	Thermal injury, penetration of the (weaker and more exposed) right hemidiaphragm
Chylous ascites	Diaphragmatic injury, PDS, lymph circulation, retrograde flow
Malignant causes	Diaphragmatic metastasis, iatrogenic injury, PDS, lymph circulation, retrograde flow
Lymph circulation abnormalities	PDS, VEGF-induced remodeling of the lymph circulation, retrograde flow

## The reverse flow – from the pleural to the peritoneal cavity

PPC occasionally manifests as reverse flow from the thorax to the abdomen, despite the average pressure gradient between them. The primary risk factor is mechanical ventilation, due to the significant increase in intra-thoracic pressure, which exceeds the intra-abdominal pressure. As a result, the reversed thoracoabdominal pressure gradient can push the pleural air or fluid to the peritoneal cavity, leading to pneumoperitoneum or ascites, respectively. In both cases, atrophy of the diaphragm is a common underlying disorder [65]. Disuse of the diaphragm induces proteolysis in the muscle fibers. Further research may be necessary to evaluate the effect of diffuse atrophy of the diaphragm in patients on mechanical ventilation and its impact on porous diaphragm syndrome and lymph circulation.

Mechanical ventilation is rarely complicated by pneumoperitoneum. These patients are affected by pneumothorax, pneumomediastinum, or both [66]. This relationship provides evidence that either pneumothorax or pneumomediastinum may result in pneumoperitoneum. It has been proposed that air passes from the mediastinum through the retroperitoneum and eventually reaches the peritoneal cavity [66, 67]. Occasionally, pleural effusion may be diminished, giving rise to ascites in the presence of PPC. In a case of patient with liver cirrhosis, a right-sided pleural effusion represented a hepatic hydrothorax, which was diminished after an unintended tension pneumothorax. Therefore, the term “bi-directional hepatic hydrothorax” was coined [68]. This only emphasizes that in cases of risk factors of PPC (either pre-existing or acquired), the movement of gas and fluids is a two-way path between the pleural and peritoneal cavity.

## Conclusions

Although the pleural and peritoneal cavities are commonly separated, communication between the two cavities is possible, under certain congenital or acquired conditions. Physicians should abandon the notion that only congenital diaphragmatic hernias and large defects are allowing for communication between the thorax and the abdomen. Although the underlying mechanisms are not always clear, PPC remains a possibly underreported condition, associated with increased morbidity and mortality and should be suspected in patients with right or bilateral pleural effusion when other causes have been ruled out.
